# Tumeurs trophoblastiques gestationnelles: aspects cliniques et radiologiques

**DOI:** 10.11604/pamj.2017.28.228.13267

**Published:** 2017-11-14

**Authors:** Nisrine Mamouni, Siham Boumhaoued, Sanae Erraghay, Meriem Boubou, Chahrazed Bouchikhi, Abdelaziz Banani

**Affiliations:** 1 Service de Gynécologie Obstétrique I, Hôpital Mère Enfant, CHU Hassan II, Fès, Université sidi Mohamed ben Abdellah, Maroc; 2 Service de Radiologie, Hôpital Mère Enfant, Hôpital Mère Enfant, CHU Hassan II, Fès, Université sidi Mohamed ben Abdellah, Maroc

**Keywords:** Tumeurs trophoblastiques gestationnelles, critères diagnostiques, polymorphisme radiologique, Gestational trophoblastic tumors, diagnostic criteria, radiological polymorphism

## Abstract

La maladie trophoblastique gestationnelle englobe un groupe de maladies qui diffèrent les unes des autres par leur évolution vers la régression, aux métastases et à la récurrence. C'est une maladie grave qui touche les femmes en âge de procréer. Les tumeurs trophoblastiques gestationnelles (TTG) représentent les formes malignes des maladies trophoblastiques gestationnelles. Elles font toujours suite à une grossesse, le plus souvent môlaire (môle hydatiforme). Le type le plus courant de tumeurs trophoblastique gestationnelle (TTG) est la mole invasive, car, dans la plupart des cas, le diagnostic est fait lorsque le cancer est encore confiné à l'utérus. Le choriocarcinome est un type plus rare qui génère souvent des métastases à distance. Dans les cas de progression vers une tumeur trophoblastique, la mise en place d'un bilan d'extension locorégional et à distance est essentielle au choix d'un protocole de traitement approprié. Les auteurs rapportent trois observations cliniques de TTG en décrivant leurs présentations cliniques et l'utilisation de techniques d'imagerie dans le diagnostic et la prise en charge de ces affections.

## Abstract (en)

Gestational trophoblastic disease incorporates a group of diseases which differ from each other by their regressive evolution, their evolution to metastasis and to recurrence. It is a severe disease that affects women of childbearing age. Gestational trophoblastic tumors (GTT) are the malignant forms of gestational trophoblastic diseases. They are always a result of pregnancy, more often molar pregnancy (hydatidiform mole). The most common type of gestational trophoblastic tumors (GTT) is the invasive mole because, in most cases, the diagnosis is made when cancer is still confined to the uterus. Choriocarcinoma is a more rare type of tumor, often developing distant metastases. When there is a progression to a trophoblastic tumor, the assessment of locoregional extension and distant metastases is essential to establish an appropriate treatment protocol. We here report three clinical cases of GTT by describing their clinical presentations and the use of imaging techniques in the diagnosis and management of these disorders.

## Introduction

Les tumeurs trophoblastiques gestationnelles (TTG) représentent les formes malignes des maladies trophoblastiques gestationnelles. Elles font toujours suite à une grossesse, le plus souvent môlaire (môle hydatiforme). Elles désignent des affections ayant en commun une sécrétion anormalement élevée et prolongée d'hormone chorionique gonadotrophique (HCG). Les gynécologues se sont longtemps méfiés de cette pathologie en raison du retard de diagnostic et des nombreux échecs d'appréciation de la gravité. Les modalités diagnostiques et de prise en charge étaient initialement basées sur l'histoire clinique et les données biologiques. Actuellement, elles incluent en grande partie l'imagerie.

## Patient et observation

**Observation 1:** Mme F.G âgée de 24 ans, G2P0, suivie pour une mole partielle en 2009 jusqu'à guérison. Après un délai de 6 ans, la patiente a consulté chez un gynécologue pour des métrorragies sur une aménorrhée de 2mois avec un taux de BHCG à 30000 UI/L. Le diagnostic de mole hydatiforme a été posé avec réalisation d'un curetage aspiratif et l'examen histologique a été en faveur de mole hydatiforme partielle. Devant la persistance des métrorragies, la patiente nous a été adressée pour prise en charge. L'examen gynécologique a objectivé la présence d'un saignement minime provenant de l'endocol et l'utérus était augmenté de taille à mi chemin de l'ombilic avec des paramètres infiltrés au toucher rectal. L'échographie pelvienne a objectivé la présence d'une masse utérine isthmique hétérogène de 67 sur 63 millimètres, hypervascularisée au Doppler couleur, semblant envahir la totalité de la paroi myometriale jusqu'à la séreuse. L'aspect échographique a été /l. L'imagerie par résonance magnétique (IRM) pelvienne a été réalisée (séquence T1, T2, diffusion, Fiesta et séquence LAVA) objectivant la présence d'un utérus volumineux faisant 16 sur 8 centimètres, siège au niveau corporeoisthmique d'un processus tissulaire intramyometrial, mal délimité, présentant en signal hétérogène hyper T1, hyper T2 partiellement restrictif en diffusion et prenant le contraste de façon précoce et intense, délimitant des zones non rehaussées, correspondant aux zones hémorragiques hyper T1 et aux zones kystiques hypo T1 et hyper T2. Ce processus mesure 89 * 89 * 75 millimètres, refoulant la ligne cavitaire et la paroi radiographie pulmonaire a objectivé la présence de quatre nodules parenchymateux. La patiente a reçu une polychimiothérapie (TTG à haut risque selon score FIGO 2000). La négativation du taux HCG sérique a été obtenue après quatre cures de chimiothérapie .L'évolution a été marquée par la survenue après la fin de la chimiothérapie de métrorragie de grande abondance motivant la réalisation d'une hystérectomie d'hémostase. A noter que les deux uretères on été prise dans la masse tumorale d'ou la décision d'une exérèse partielle avec une réimplantation urétérale. L'étude anatomopathologique en faveur d'un utérus siège de remaniements hémorragiques.

**Observation clinique 2:** Mme KH, âgée de 30 ans, sans antécédents pathologiques notables, troisième geste troisième pare, la dernière grossesse n'était pas suivie, menée à terme, avec un accouchement par voie basse d'un nouveau né eutrophique, âgé actuellement de 3 ans, puis elle a été mise sous contraception hormonale. La patiente ayant consulté aux urgences de maternité pour un saignement vaginal de moyenne abondance persistant depuis 4 jours sans notion d'aménorrhée. A l'admission, l'examen au speculum trouve un saignement provenant de l'endocol, avec la présence de plusieurs lésions vaginales exophytiques irrégulières, de taille infracentimetrique, au toucher vaginal, le col est de consistance normale avec un utérus qui arrive à mi chemin de l'ombilic. L'échographie pelvienne a objectivé la présence d'une image tissulaire utérine de 65 millimètres prenant le doppler de siège isthmique interrompant la ligne d'interface. Le taux de HCG totale sérique est de 1200000 UI/L. Le diagnostic de TTG a été fortement suspecté. L'IRM pelvienne ayant objectivé la présence d'un processus tissulaire isthmocorporeal de signal hétérogène envahissant le myomètre sur toute son épaisseur et s'étendant vers le vagin et les grandes La TTG a été classée à haut risque selon le score FIGO 2000 d'où la décision d'administration d'une polychimiothérapie.

**Observation 3:** Mme DR, âgée de 45 ans, neuvième geste huitième pare, admise pour prise en charge de grossesse molaire. Le taux de HCG sérique à 200000 UI/L. un curetage aspiratif a été réalisé jusqu'à obtention de vacuité utérine puis la patiente a été perdue de vue ayant reconsulté un mois après avec des métrorragies persistantes. Le taux HCG de contrôle revenant à 1000000UI/L. Une échographie pelvienne réalisée objectivant la présence d'un processus tissulaire endouterin envahissant le myomètre et arrivant jusqu'à la séreuse, très vascularisé au Doppler couleur avec a objectivé la présence d'un processus tissulaire endouterin envahissant le myomètre jusqu'à la séreuse sans extension extra-utérine et une thrombose de la veine ovarique droite s'étendant jusqu'à la veine cave plusieurs nodules parenchymateux pulmonaires infra centimétriques. Nous n'avons pas réalisé d'IRM pelvienne par manque de moyens. La patiente a été scorée à haut risque d'où l'indication d'une polychimiotherapie. Vu la persistance des métrorragies continues de faible abondance, une embolisation des artères utérines a été réalisée. La patiente a accusé des douleurs abdominales aigues 48 heures après, d'où la réalisation d'une échographie abdominale objectivant la présence d'un épanchement intraperitoneal de grande abondance. La patiente a été acheminée en urgence au bloc opératoire où une laparotomie fut réalisée au cours de laquelle un hémopéritoine de deux litres a été aspiré avec la réalisation d'une hystérectomie d'hémostase en passant au large de la tumeur (celle ci ayant envahi la séreuse utérine avec extension vers les paramètres) suivie d'une résection partielle et réimplantation de l'uretère pelvien droit qui était entouré de vésicules trophoblastiques (à signaler que l'envahissement parametrial droit n'a pas été objectivé au scanner pelvien). L'étude anatomopathologique est revenue en faveur d'une mole invasive.

## Discussion

**Figure 1:**
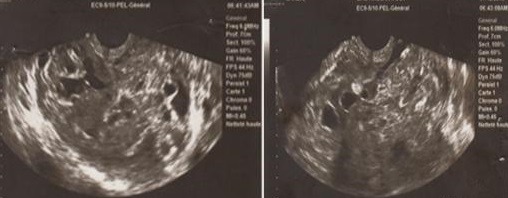
Échographie endovaginale; coupe sagittale: processus tissulaire endocavitaire envahissant le myomètre, hétérogène, mal délimité

**Figure 2:**
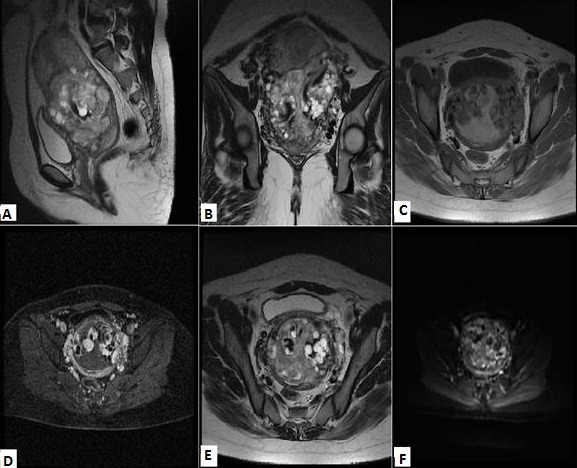
IRM pelvienne; A) coupe sagittale T2: processus tissulaire intra-myomètrial multivésiculaire, en hypersignal hétérogène, franchissant la séreuse; B) coupe coronale T2, 2 lésions tissulaires hétérogènes en hypersignal; C) coupe axiale T1 (C-): 2 processus tissulaires hétérogènes en intra utérin; D) coupe axiale T1 (C+): prise de contraste précoce et intense, délimitant des zones non rehaussées correspondant aux zones hémorragiques en hypersignal et aux zones kystiques en hyposignal; E) coupe axiale T2, infiltration de la graisse paramétriale bilatérale; F) diffusion b600: les deux lésions en signal hétérogène, partiellement restrictif en diffusion

**Figure 3:**
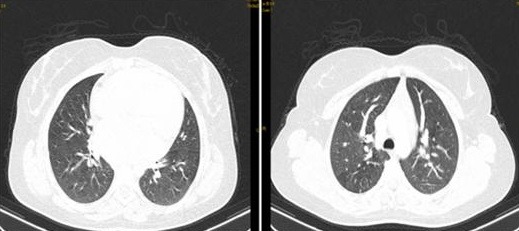
Scanner thoracique; coupe axiale: présence de plusieurs nodules bilatéraux pulmonaires de tailles variables

**Figure 4:**
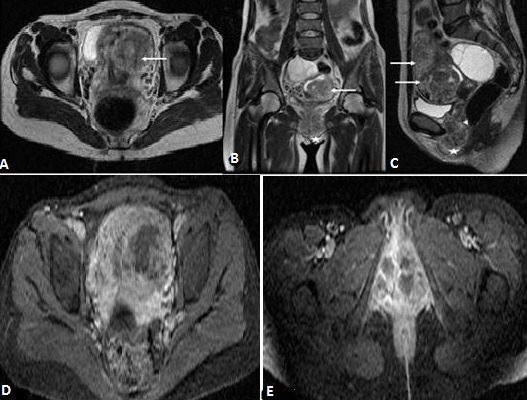
IRM pelvienne, séquence T2, les plans axial (A), coronal (B) et sagittal (C), processus tissulaire utérin envahissant le myomètre (flèche), envahissement le vagin (tête de flèche), et les grandes lèvres (astérix); D et E) séquence T1 après injection du gadolinium et saturation du signal de la graisse dans le plan axial: prise de contraste hétérogène avec délimitation de zones nécrotiques non rehaussées

**Figure 5:**
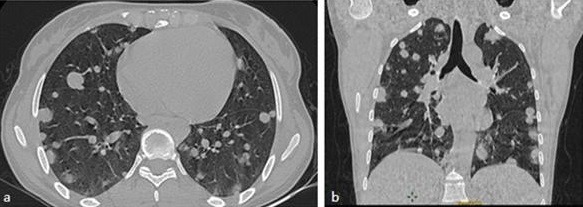
TDM thoracique faite dans le cadre du bilan d’extension en coupe axiale (a) et reconstruction coronale (b) objectivant la présence de multiples nodules pulmonaires intéressant les différents lobes sans prédominance topographique définissant ainsi un aspect en lâcher de ballon

**Figure 6:**
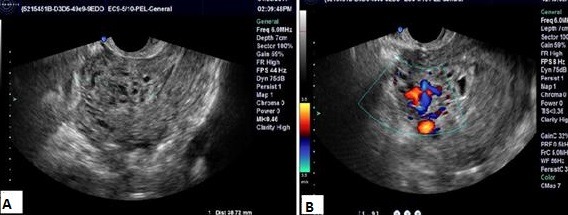
Échographie pelvienne; coupe sagittale: processus tissulaire endo-utérin envahissant le myomètre jusqu’à la séreuse, hétérogène, richement vascularisé au Doppler couleur (prise du signal Doppler couleur au niveau des zones anechogenes)

**Figure 7:**
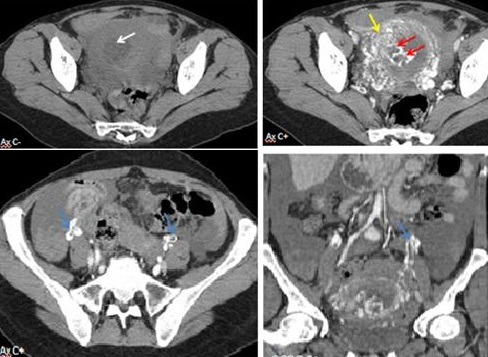
TDM pelvienne; masse endo-cavitaire spontanément hypodense (flèche blanche) micro-kystique hyper-vascularisée (flèche rouge) envahissant le myomètre (flèche jaune); thrombose partielle des veines ovariques bilatérales (flèche bleue)

Les maladies trophoblastiques gestationnelles comprennent un large spectre de pathologies du placenta, allant des lésions bénignes précancéreuses (les moles hydatiformes complètes ou partielles), aux néoplasies trophoblastiques gestationnelles ou TTG. Les maladies trophoblastiques sont en général l'apanage des femmes en période d'activité génitale, les formes malignes des maladies trophoblastiques gestationnelles. Elles font toujours suite à une grossesse, le plus souvent môlaire (môle hydatiforme). Elles désignent des affections ayant en commun une sécrétion anormalement élevée et prolongée de l'hormone chorionique noter la persistance ou la réapparition des métrorragies, accompagnées parfois d'une altération progressive de l'état général avec asthénie, anorexie et amaigrissement. Dans le cadre des symptômes paranéoplasiques, (dyspnée, Hypertension intracranienne ). Cependant, parfois la patiente est au début asymptomatique. Sur le plan biologique, la présence d'une TTG va se révéler par une évolution perturbée de la courbe de HCG, après une grossesse molaire. Après une grossesse non molaire, une tumeur utérine persistante sécrétant de l'HCG correspond presque toujours à un carcinome trophoblastique (le cas de l'observation 2 où le diagnostic de TTG polymetastatique a été posé trois années après une grossesse menée à aginale peut objectiver la présence des zones hypoéchogènes et nodules intramyometriaux, avec des zones hypoéchogènes dans l'endomètre (lacunes vasculaires) entourées de zones hyperéchogènes (nodules trophoblastiques). Les images de mole invasive sont en général diffuses et hétérogènes et contiennent souvent des images par resonance magnetique (IRM) pelvienne permet de dresser un bilan d' extension locorégionale. L'IRM pelvienne est utile à l'évaluation de l'extension myometriale et pelvienne de la TTG. Les aspects IRM ne sont pas spécifiques car seule la biopsie permet d'établir un diagnostic histologique précis. La mole fortement évocateur d'une TTG (Mole invasive ou choriocarcinome) (Figure 1). Un contrôle du taux de HCG sérique est revenu positif à 123885 UI postérieure du myomètre et envahissant la séreuse utérine (Figure 2). Pour évaluer l'extension à distance, un scanner cerebrothoracoabdominal a été réalisé, objectivant la présence de localisations pulmonaires secondaires (Figure 3). Une lèvres (Figure 4). Un scanner cérébrothoracoabdominal a objectivé la présence de lésions pulmonaires, spléniques et rénales d'allure secondaire (Figure 5). présence d'un épanchement intra péritonéal de faible abondance (Figure 6). Le bilan d'extension fait de scanner cérébro-thoraco-abdomino-pelvien inferieure correspondant probablement à des embols tumoraux vasculaires (Figure 7). Le scanner thoracique a objectivé la présence de mais restent décrites lors de la périménopause et ménopause [1]. Les tumeurs trophoblastiques gestationnelles (TTG) représentent gonadotrophique (HCG). Il existe principalement quatre types de TTG qui sont La môle invasive, le choriocarcinome, la tumeur du site d'implantation placentaire, la tumeur trophoblastique épithéloide [2]. Sur le plan clinique la symptomatologie est très polymorphe [3]. Le signe le plus fréquent est les métrorragies. Généralement c'est au cours de la surveillance clinique d'une môle hydatiforme, où on peut dans la littérature, une hyperthyroïdie est notée dans 3 à 5% des cas. Elle est sans doute en rapport avec l'effet thyréotrope de l'HCG [4]. Il existe aussi des cas où les métastases inaugurent le tableau clinique terme) [3]. Les critères diagnostiques retenus sont ceux proposés en 2000 par la FIGO. Ils reposent sur un consensus d'experts [5]. Une fois le diagnostic de TTG posé, il est recommandé d'en évaluer l'extension, qui conditionne le prognostic. Les centres de référence français préconisent une harmonisation du bilan d'extension [5]. Pour l'evaluation de l'extension locale, il est recommandé de réaliser une échographie pelvienne endovaginale, si possible accompagnée d'un doppler couleur. Les différents types des TTG peuvent apparaitre similaires à l'échographie [6]. L'échographie endov kystiques [7-10]. La taille initiale de la tumeur utérine mesurée par échographie est un facteur pronostique [11]. Une hyper vascularisation de certaines zones échographiquement hétérogènes du myomètre contribue à la localisation et au diagnostic de mole invasive [12-14]. L'imagerie invasive et le choriocarcinome correspondent le plus souvent à une masse hétérogène myometriale en séquence T1 et T2 [15]. Peu de cas de TSI ont été décrits dans la littérature. L'aspect en IRM et celui d'une masse solide myometriale avec un signal d'intensité kystiques [16-18]. Le scanner est le meilleur moyen d'imagerie pour évaluer les différents sites métastatiques des TTG [1], excepté pour les métastases cérébrales et vaginales. Une variété de site métastatique a été rapportée, tel le foie, la rate, les reins, tube digestif et cerveau. Les métastases ganglionnaires sont plus fréquentes en cas de tumeur du site d'implantation [19-21]. Le scanner thoracique permet la recherche de métastases pulmonaires, que si hétérogène, une vascularisation tumorale très développée avec la présence de zones elles sont présentes, ceci conduira à la réalisation d'une radiographie pulmonaire afin de les dénombrer et les mesurer. Le scanner abdominal recherchera des métastases hépatiques et les métastases cérébrales sont recherchées par IRM cérébrale ou à défaut par scanner.

## Conclusion

Le diagnostic des TTG repose sur l'histoire clinique confrontée aux données des examens complémentaires. L'imagerie des TTG n'étant pas spécifiques, néanmoins le bilan radiologique aide au diagnostic via l'échographie pelvienne en première intention et permet l'élaboration du score FIGO dictant une conduite thérapeutique même en l'absence de preuve histologique.

## Conflits d’intérêts

Les auteurs ne déclarent aucun conflit d’intérêts.
